# HIV-infected adolescents have low adherence to antiretroviral therapy: a cross-sectional study in Addis Ababa, Ethiopia

**DOI:** 10.11604/pamj.2017.27.80.8544

**Published:** 2017-06-02

**Authors:** Naod Firdu, Fikre Enquselassie, Degu Jerene

**Affiliations:** 1 Addis Ababa University, School of Public Health, Department of Preventive Medicine, Addis Ababa, Ethiopia; 2 Management Sciences for Health (MSH), Addis Ababa, Ethiopia

**Keywords:** Antiretroviral therapy, adherence, adolescent, patient self-report, Addis Ababa, Ethiopia

## Abstract

**Introduction:**

For antiretroviral therapy (ART) to work effectively, adherence is very crucial. However, most studies done on ART adherence are either on children or on adults. There is limited information on the level of adherence among adolescents.

**Methods:**

Using a cross-sectional study design, we interviewed 273 HIV-infected adolescents receiving ART from three hospitals in Addis Ababa. We used a structured questionnaire to measure adherence levels using patient self-reports. Bivariate and multivariate methods were used for analysis.

**Results:**

We interviewed 273 adolescents aged 13 to 19 years, and 144 (52.7%) of the participants were girls. Their mean age was 15.4 years (SD± 1.75). The self-reported adherence rate of the respondents was 79.1% (216/273). On bivariate analysis, variables like WHO clinical stage, being on Cotrimoxazole Prophylactic Therapy (CPT), marital and living status of the parent, whether parent was on ART or not and having special instructions for ART medications were associated with optimum adherence. However of those, only WHO stage IV (adjusted OR, 12.874 95% CI, 2.079-79.706), being on CPT (adjusted OR, 0.339 95% CI, 0.124-0.97) and adolescents with widowed parent (adjusted OR, 0.087 with 95% CI, 0.021-0.359) were found to be significantly associated with optimum ART adherence.

**Conclusion:**

The level of self-reported ART adherence among HIV-infected adolescents at the three hospitals was below the recommended threshold. Though earlier presentation of adolescents to care should be encouraged, more targeted adherence support should be planned for those who present at an early stage of their illness.

## Introduction

Adolescence is the period between 10 and 19 years of age with about one-fifth of the world's population belonging to this age group [1]. In the sub- Saharan Africa (SSA) including Ethiopia, one-third of the total population is aged between 10 to 24 years [2]. Adolescence is a period of dynamic change representing the transition from childhood to adulthood. During this stage, rapid changes in physical, emotional, cognitive and social characteristics take place [1]. In many resource-poor settings, adolescents are now the emerging group of HIV-infected population as survival of children with perinatally acquired HIV infection into adolescence is increasingly being documented [3, 4]. In addition to the perinatally acquired infection, adolescents and young adults are vulnerable to acquiring HIV through sexual route [4]. Eighty-two percent of the estimated 2.1 million adolescents aged 10-19 years living with HIV by the end of 2012 were in sub-Saharan Africa [5]. Though, less than one percent of Ethiopian youth tested positive for HIV on the 2011's Ethiopian Demographic and Health Survey (EDHS), according to the regional estimates of HIV prevalence among youth, Addis Ababa (capital of Ethiopia) has the second highest prevalence next to Gambella region [6]. Similarly, HIV is significantly prevalent among youth in Addis Ababa, particularly among out- of school and female youth [7]. This suggests that there is a need to pay attention to factors affecting access to and outcomes of treatment among adolescents and young adults. The most important factor which determines the success of ART is sustainable and optimum adherence to therapy [8] as poor adherence is associated with treatment failure and the development of viral resistance [9]. Adherence to medication has been described as the proportion of prescribed medications that is actually taken. It is measured on a scale from zero to 100%. The recommended optimal adherence level for ART to be effective is above 95 percent [10]. However, there is limited information on the levels of adolescent ART adherence in resource-poor settings [11]. Studies done on ART adherence in Ethiopia are either on children or on adults and those showed high adherence rates [12, 13]. Studies on the level and predictors of ART adherence among adolescents in Ethiopia are lacking. Therefore, in this study, we aimed to contribute to filling this information gap.

## Methods

**Study area**: The study took place in three hospitals in Addis Ababa City, the Capital City of the Federal Democratic Republic of Ethiopia. It was carried out in the ART units of Tikur Anbessa, Saint Paul, and Zewditu Memorial hospitals which were selected based on high case load. Tikur Anbessa Hospital is the largest teaching hospital in Ethiopia; Saint Paul is a General Specialized Teaching Hospital; while Zewditu is a Referral Hospital with the largest number of ART patients in Addis Ababa. All the three hospitals have separate ART clinics for children and adults. Adolescents are cared for in either the child or in the adult clinics. There are no separate adolescent ART clinics. The study period was from July 2013 to June 2014.

**Study design**: The study used a facility based cross-sectional study design. All HIV-infected adolescents in Addis Ababa constituted the target population. The study population included all adolescents who were taking ART and on follow-up in the selected hospitals during our study period.

**Sample size and selection of study participants**: We calculated the sample size using StatCalc of Epi info version 3.5.4 software package by considering the following assumptions: Proportion of non-adherence among unexposed, 23.1%; 80% power; 95% confidence interval; Odds ratio of 2 and 10% Non-response rate. Since this study focused on a unique population of limited size, the finite population correction is applied and the final sample size was n= 303. This was allocated proportionally to the three facilities after determining the population size in each facility.That is total number of adolescents on HAART at each institution. Using the ART registers and site electronic database, we prepared a list of all adolescents aged between 13-19 years and who were on ART as a sampling frame. We then selected study participants using systematic random sampling technique.

**Study variables**: The dependent variable in this study was 'adherence to ART' and independent variables included socio-demographic, behavioural and clinical factors. A participant was said to have optimal ART adherence if he or she took ≥ 95% of the prescribed pills correctly for the four days prior to the study.

**Data collection tools and procedures**: We used a structured, pre-tested questionnaire which was developed based on a review of various literature. The questions were divided into four sections: (i) socio-demographic characteristics, (ii) clinical information, (iii) adherence questions and (iv) behaviour related questions. To measure adherence we adapted questions from AIDS Clinical Trials Group (ACTG) which assesses the ART adherence for the four days prior to the survey [14]. We recruited nurses working in the ART clinics of the selected facilities as interviewers. We then provided them with two days training on the study and on the standard operating procedure. We pre-tested the tool on non-sampled adolescents and modified it accordingly. Nurses collected data by face to face interview with the adolescents in a private room. For adolescents whose HIV status was not disclosed, the nurses took data regarding ART medications adherence from parents or caregivers. We used patient medical charts to retrieve clinical information.

**Data entry and analysis**: We used Epi Info version 3.5.4 for data entry and SPSS version 21 for analysis. First, we did descriptive statistics to explore the socio-demographic characteristics of the respondents, the adherence rate and clinical characteristics of the adolescents. Then, we explored for the association between the various independent variables with optimum ART adherence on bivariate analysis. We then identified those variables which were associated with adherence at a level of P-value of less than 0.05. We included those variables in multivariable analysis.

**Ethical considerations**: The School of Public Health at Addis Ababa University and the Institutional Review Boards of the hospitals reviewed and approved the research protocol. We sought permission to conduct the study from the Medical Directors of the three hospitals. Adolescents and caregivers received information about the study. We obtained written informed consent from adolescents aged 18 and above. For adolescents under 18 years, we obtained consent from parents or legal guardians in addition to verbal assent by the young. To ensure confidentiality of all study participants, we used no direct identifiers in the data collection, storage or report writing. All electronic documents were password protected and all paper documents were stored in a locked cabinet. We prevented accidental disclosure of HIV status to those non-disclosed adolescents. This was dealt with by training the interviewer on the study protocol and by collecting data regarding adherence from parent or caregiver.

## Results

**Socio-demographic Characteristics of Participants**: We interviewed 273 adolescents aged 13 to 19 years with a response rate of 91%, and 52.7% of them were girls. The mean age of the participants at study enrolment was 15.4 years (SD ± 1.7). Nearly all (97.8%) were living in urban settings. One-third of the adolescents had both parents dead and 39 (14.3%) were cared for by their grandparents. The majority 165 (60.5%) of the adolescents were cared for by either of their parents. Only 45 (16.5%) of the caregiver's had external monetary support for the adolescent. Table 1 provides the socio-demographic characteristics of the study participants.

**Table 1:** Socio-demographic characteristics of adolescents on ART Addis Ababa, Ethiopia 2014; (n=273)

Characteristics (variable)	Frequency	Percentage
**Sex of adolescent**		
Male	129	47.3%
Female	144	52.7%
**Age of adolescent (at study enrolment)**		
13-14 years	96	35.2%
15-17 years	141	51.6%
18-19 years	36	13.2%
**Mean Age: 15.4 with SD: 1.7**		
Urban	267	97.8%
Rural	6	2.2%
**Caregivers relation with adolescent**		
Mother	96	35.2%
Father	69	25.3%
Brother	6	2.1%
Sister	15	5.5%
Grandparent	39	14.3%
Other relative	48	17.6%
**Parents living status**		
Both parents alive	90	33%
Father is dead	51	18.7%
Mother is dead	54	19.8%
Both parents are dead	78	28.6%
**External monetary support**		
No support	228	83.5%
supported	45	16.5%

**Behavioural characteristics of adolescents on ART**: Out of the 273 adolescents interviewed, 9(3.3%) have tried to smoke a cigarette. Among those, the earliest age when a whole cigarette was smoked was 14years, 3 out of the 9 did at this age. None of the nine did smoke cigarette daily. Similarly, 9(3.3%) of the respondents have used Khat (stimulant leaf) and all of those nine were male respondents. Concerning alcohol use, 21(8%) of the participants have used alcohol in the thirty days prior to the survey. Of the 21, three of the respondents used alcohol on a daily basis for the thirty days prior to the study. Regarding sexual behaviour, 12(4.4%) of the study participants have had sexual intercourse. Of the 12, three used substance while having the last sexual intercourse; six did not use a condom at the last sexual act. The earliest age at first sex was 14 years.

**Clinical characteristics**: Close to three-fourth of respondents 192(71.2%) were either in WHO clinical stage III or IV at the initiation of ART, and 45.1% had CD4 count less than or equal to 200 cells per ml. Half of the participants 135 (49.5%) were on cotrimoxazole prophylactic therapy (CPT) at the time of the study. Most (93%) of the adolescents were having HIV care and follow up at paediatric ART clinics, and 90.1% of the respondents were informed of their HIV status. The median haemoglobin level at baseline was 12.3gm/dl (range: 8.5-17.9). Looking at the health facilities where the adolescents were enrolled in; 134 (49.1%) were in Zewditu Memorial Hospital, 94(34.4%) were in Tikur Anbessa Hospital and the remaining in Saint Paul Hospital. Hundred and twenty-nine (71.7%) of the parents were enrolled in HIV care and treatment and 141 (81%) of parents have disclosed their HIV status. Close to a half of the participants 123(45.1%) had a history of hospital admission before starting ART. Table 2 below describes the clinical characteristics of adolescents on ART.

**Table 2:** Clinical characteristics of adolescents on ART in Addis Ababa, Ethiopia 2014

Variable	Number (frequency)	Percentage
**WHO clinical stage at baseline^#^**		
Stage I	21	7.8%
Stage II	57	21%
Stage III	123	45.6%
Stage IV	69	25.6%
**Baseline CD4 count**		
<100	30	11%
100-200	93	34.1%
201-500	117	42.9%
>500	33	12%
**On CPT (at study enrolment)**		
Yes	135	49.5%
No	138	50.5%
**ART follow-up clinic**		
Paediatric	254	93%
Adult	19	7%
**Adolescent HIV status Disclosure**		
Disclosed	246	90.1%
Not disclosed	27	9.9%
**Baseline Haemoglobin** **(Median)**	12.3gm/dl	
**Pre-ART hospital admission**		
Yes	123	45.1%
No	150	54.9%
**Parental HIV status Disclosure**		
Disclosed	141	81%
Not disclosed	33	19%
**Parent enrolled in HIV care**		
Enrolled	129	71.7%
Not enrolled	51	28.3%

**Adherence and other features related to ART drugs**: The vast majority of the adolescents 252 (92.3%) started ART in the facilities where they were enrolled at the time of this study. Almost all were on first line medications 262(96%). Hundred and forty-seven (53.8%) had either of their parents on ART at the time of the survey. The median duration on ART was 7 years (IQR= 3) whereas the mean age at ART initiation was 8.85 years (SD of 2.661years). The self-reported adherence rate of the respondents for the four days was 79.1% (216/273). Thirty-nine (14.3%) of the respondents reported missing full day's medication within the prior four days. Six (2.2%) of the adolescents missed all ART doses of the four days i.e. zero adherence, and 22% of the participants missed, at least, a single pill. Hundred and fourteen (41.8%) reported following specific medication schedule all of the time; seventy-five (27.5%) reported following the schedule most of the time for the four days. One hundred fifty (54.5%) reported having ART medication with special instructions. In 99 (55.9%) of the cases, parents were enrolled for HIV care at a different health facility than the adolescent. Table 3 depicts adherence and other characteristics related to ART drugs among adolescents.

**Table 3:** Adherence and other characteristics related to ART drugs among HIV-infected adolescents in Addis Ababa, Ethiopia 2014

Variable	Number (frequency)	Percentage
**ART regimen**		
First line	262	96%
Second line	11	4%
**Parent on ART**		
Yes	147	53.8%
**No**	126	46.2%
**Duration on ART in years**		
< 6 years	75	27.5%
6 years and above	198	72.5%
**Adolescent ART adherence**		
Adherent (≥95%)	216	79.1%
Non-adherent (< 95%)	57	20.9%
**Missed full day’s medication**		
Yes*	39	14.3%
No	234	85.7%
**Special Instruction with ART**		
Yes	150	54.5%
No	123	45.5%
**Parent Enrolment**		
Same facility	48	27.1%
Different facility	99	55.9%
Not known	30	16.9%

* In one or more of the four day’s prior the study, adolescent missed full day’s medication

**Socio-demographic features of parents or caregivers**: The median age of caregivers was 42 years. Over a half (60%) of the caregivers were either single or were widowed while 38.9% were married. Concerning the educational status of the caregivers, 54(19.8%) had some university education; 90(33%) completed or had some secondary education. Looking at the occupation of the caregivers, 88 (31.9%) had private jobs, 67 (24.5%) were housewives, 34 (12.5%) were government employees and 11.7% of the caregivers were unemployed. The median number of caregivers. children was 2 (range: 0 to 9).

**Factors associated with optimal ART adherence**: Table 4 Describes factors associated with optimal ART adherence on bivariate and multivariate analyses. On bivariate analysis, we examined whether various explanatory vvariables are associated with optimal adherence, considering a P-value of less than 0.05 as a cut-off for statistical significance. We found six variables namely; baseline WHO clinical stage, being on CPT, caregiver's marital and living status, whether parent was on ART and special instruction with ART medications to have a significant association with optimal ART adherence. We then conducted a multivariate analysis controlling for those six covariates to adjust for possible confounder effect. The multivariate analysis yielded; WHO stage IV (adjusted OR, 12.874 95% CI, 2.079-79.706), being on CPT (adjusted OR, 0.339 95% CI, 0.124-0.97) and adolescents with widowed parent (adjusted OR, 0.087 with 95% CI, 0.021-0.359) to have an independent and statistically significant association with optimum ART adherence.

**Table 4:** Factors associated with optimal ART adherence on bivariate and multivariate analysis

Variable	Adherence level(≥ 95% adherent)	P-value *(bivariate)*	Crude Odds ratiowith 95% CI *(bivariate)*	Adjusted ORWith 95% CI *(multivariate)*
**WHO clinical stage**				
Stage I	13/21 (61.9%)	(reference)		
Stage II	40/57 (70.2%)	0.489	1.448 (0.508-4.128)	2.624 (0.631-10.918)
Stage III	96/123 (78%)	0.117	2.188 (0.822-5.823)	1.449 (0.419-5.009)
Stage IV	64/69 (92.75%)	0.001*	7.877 (2.220-27.950)	12.874 (2.079-79.706)*
**On CPT**				
Yes	97/135(71.85%)	0.004*	0.408 (0.221-0.752)	0.339 (0.124-0.97)*
No	119/138(86.2%)	(reference)		
**Parent marital status**				
Married	94/105 (89.5%)	(reference)		
Single	67/81 (82.7%)	0.181	0.560 (0.239-1.310)	0.528 (0.141-1.980)
Widowed	52/81 (64.2%)	0.0001*	0.210 (0.097-0.454)	0.087 (0.021-0.359)*
**Parent living status**				
Both are alive	82/90 (91.1%)	(reference)		
Father is dead	34/51 (66.67%)	0.001*	0.195 (0.077-0.495)	1.044 (0.244- 4.466)
Mother is dead	34/54 (62.96%)	0.0001*	0.166 (0.067-0.413)	0.601 (0.147-2.464)
Both are dead	66/78 (84.62%)	0.200	0.537 (0.207-1.390)	5679 (.000-
**Parent on ART**				
Yes	118/147 (80.2%)	0.039*	2.3 (1.042-5.078)	1.196 (0.353-4.050)
No	23/36 (63.89%)	(reference)		
**Special medication instruction**				
Yes	26/150 (84%)	0.03*	1.925 (1.066-3.477)	1.387 (0.567-3.396)
No	90/123 (73.2%)	(reference)		

## Discussion

In this study, we assessed the magnitude of ART adherence among HIV-infected adolescents in Addis Ababa and we also looked into the factors associated with optimal ART adherence. We found the ART adherence level to be lower than the recommended level and less advanced disease stage, taking CPT along with ART and those cared for by widowed parents were significantly associated with poorer adherence levels. The level of non-adherence (20.9%) is quite high and puts the adolescents at higher risk of drug resistance and treatment failure. Also, of great concern is that significant proportion of the respondents reported missing full day's medication of one or more days in the four days prior to the study. Furthermore, six of the adolescents had zero percent adherences; did not take any ART medication in the four days. This suggests the need for more targeted adherence interventions for adolescents in early disease stage, those on concomitant medications and adolescents living with widowed parents. The low self-reported adherence in our study is in line with other study findings. A study was done among American adolescents aged 12-18 years revealed low ART adherence level [15]. This study, unlike ours, longitudinally followed a cohort of 231 HIV + adolescents. Though it used a similar self-report method to assess adherence it was further validated by various additional ways. Another study done in Botswana came up with adolescent ART adherence rate of 76.9%. Despite a small sample size, this study was done in a similar group of patients, 13-20 years and used patient self-reports method [16]. Similarly, other studies as well showed low ART adherence among adolescents [17–19]. In contrast to these a study done in Gaborone, Botswana reported that high proportion of the studied adolescents had excellent ART adherence. This study considered excellent adherence using pill count method when greater than or equal to 95% of the prescribed doses for one month were taken by the end of the month. However, in this study the investigators used a smaller sample size and the study was done in a single health facility [20]. A systematic review of 50 articles involving 10725 adolescents on ART reported overall adherence level to be 62.3%. In this study, ART adherence level for Africa was found to be 84% which is slightly higher than our finding of 79.1% [21]. Most of the studies on ART adherence in Ethiopia are either on children or on adults. A study in Addis Ababa assessed the ART adherence among children and found a higher adherence level, 86.9% [12]. Similarly, a prospective study of adult HIV patients in Ethiopia found a higher ART adherence rate, 94.3% [13]. Contrary to these findings a study which measured adherence using unannounced home-based pill count revealed a very low adherence level, 34.8%. This study was done among HIV-infected children below 15 years who were attending paediatric ART clinic of Tikur Anbessa Hospital [22].

The poorer adherence level among adolescents in earlier disease stage is consistent with finding from other studies. A study in Ethiopia revealed that children and adolescents in WHO stage III/IV were more likely to adhere [22]. This could be because those who are relatively healthy will be reluctant about taking their medications. Gibbs study revealed that symptomatic HIV disease was associated with better adherence [23]. Catz et al also found that healthy HIV-infected out patients had lower rates of adherence to medical appointments than the symptomatic ones [24]. A study in Uganda also found that those who had been hospitalized two or more times had better adherence [25]. On the contrary, the previously mentioned American cohort study showed that those with late HIV disease stage were less likely to be adherent compared with those in the early stage of the disease [15]. The poorer adherence among adolescents living with widowed parents could be because married parents tend to be emotionally and economically better and might also get support from their partner in giving care and support to the adolescent. Moreover, this may also be because the adolescent is living with a single parent who may be dealing with his or her own HIV status or could be too sick to take care of the adolescent. On the other hand, poorer adherence among those who were on CPT could be due to high bill burden but a false sense of security with CPT or even misunderstanding CPT as a replacement for ART could be another factor. However a study in Addis Ababa described that those who took CPT besides ART were more than three times likely to adhere than those who didn't (OR = 3.65 with 95% CI, 1.24-10.74) [12]. Accurate measurement of adherence to therapy is oftentimes difficult. There are different ways of adherence assessment including; patient self-reports, pill count method, biochemical assays of drug levels and electronic monitoring system. All of these techniques have their own limitations. A major limitation of self-reports is that they assess only short-term adherence and may often overestimate it. Moreover, this method assumes that patients can correctly recall their behaviour and are providing honest answers. Pill count method measures adherence by counting the returned excess pills which should have been taken. Here patients are expected to return the excess pills on their refill visit date. Similar to the former this method tends to overestimate adherence as patients tend to discard the package inadvertently. In addition, some patients may also discard packages purposively to appear adherent. The latter two techniques, assays of drug levels and electronic monitoring system, tend to be sophisticated and costly [26]. Our study has some limitations. First would be the use of ART nurses to collect data on medication adherence. This might introduce social desirability bias and lead to under-reporting of non-adherence by adolescents. However, we used ART nurses because we wanted to keep sensitive HIV-related adolescent information confidential. Secondly, for a small group of adolescents whose HIV status was not disclosed, 27 (9.9%), nurses took data regarding ART medications adherence from parents or caregivers. There is a chance that caregivers may not accurately recall adolescents' adherence information. However, we used them with the intention of preventing accidental disclosure of HIV status to those non-disclosed adolescents. Thirdly, with regard to selecting study facilities, we purposefully chose three public hospitals in Addis providing ART and HIV care services for a large number of HIV-positive adolescents in the city. These hospitals, however, are not the only sites providing ART for adolescents in Addis Ababa.

## Conclusion

The findings of this study indicated that the ART adherence rate among adolescents in Addis Ababa is low. Advanced WHO stage and having a married parent were associated with better ART adherence. On the other hand being on CPT was associated negatively with ART adherence. Health care providers should strengthen adolescent ART adherence counselling services in the ART clinics. Though earlier presentation of adolescents to care should be encouraged, more targeted adherence support should be planned for those who present at an early stage of their illness. In addition, adolescents or their guardians should be provided with adequate counselling during medication change or when new medication with different role is added. Further studies should assess the reasons for low adherence and to come up with more concrete interventions. Future studies should employ more than one adherence measurement tools using longitudinal study designs to avoid overestimation and help to assess the adherence over a longer period of time.

## What is known about this topic

- Most studies on ART adherence focus either on adults or on children especially in Ethiopia and other sub-Saharan countries;
- There is limited data on the level of ART adherence among adolescents in low resource countries.

## What this study adds

- Provides evidence on the level of ART adherence among HIV infected adolescents in Addis Ababa, Ethiopia;
- It shows that HIV infected adolescents in Addis Ababa have low level of adherence to antiretroviral therapy.

## Competing interests

The authors declare no competing interests.
